# Increased intramuscular adipose tissue of the quadriceps at admission is more strongly related to activities of daily living recovery at discharge compared to muscle mass loss in older patients with aspiration pneumonia

**DOI:** 10.1186/s12877-024-04718-7

**Published:** 2024-01-29

**Authors:** Naoki Akazawa, Keita Funai, Toshikazu Hino, Ryota Tsuji, Wataru Tamura, Kimiyuki Tamura, Akemi Hioka, Hideki Moriyama

**Affiliations:** 1 Department of Physical Therapy, Faculty of Health and Welfare, Tokushima Bunri University, Hoji 180, Nishihama, Yamashiro-Cho, Tokushima-City, Tokushima, 770-8514 Japan; 2 Department of Rehabilitation, Kasei Tamura Hospital, Wakayama, Wakayama Japan; 3 Life and Medical Sciences Area, Health Sciences Discipline, Kobe University, Kobe, Hyogo Japan

**Keywords:** Intramuscular adipose tissue, Aspiration pneumonia, Activities of daily living, Muscle mass, Quadriceps

## Abstract

**Background:**

Recent studies reported that an increase in intramuscular adipose tissue of the quadriceps in older patients negatively affects the recovery of activities of daily living (ADL) more than the loss of muscle mass. However, whether intramuscular adipose tissue of the quadriceps in older patients with aspiration pneumonia is related to ADL recovery remains unclear. This study aimed to determine the relationship between intramuscular adipose tissue of the quadriceps and ADL recovery in older patients with aspiration pneumonia.

**Methods:**

Thirty-nine older inpatients who were diagnosed with aspiration pneumonia participated in this prospective study. The main outcome of this study was ADL at discharge. ADL were assessed using the Barthel Index (BI). The intramuscular adipose tissue and muscle mass of the quadriceps were evaluated at admission using echo intensity and muscle thickness observed on ultrasound images. A multiple linear regression analysis was performed to confirm whether the quadriceps echo intensity was related to the BI score at discharge, even after adjusting for confounding factors.

**Results:**

The medians [interquartile range] of the BI score at admission and discharge were 15.0 [0.0–35.0] and 20.0 [5.0–55.0], respectively. The BI score at discharge was significantly higher than that at admission (*p* = 0.002). The quadriceps echo intensity (β =  − 0.374; *p* = 0.036) and BI score at admission (β = 0.601; *p* < 0.001) were independently and significantly related to the BI score at discharge (R^2^ = 0.718; f^2^ = 2.546; statistical power = 1.000). In contrast, the quadriceps thickness (β =  − 0.216; *p* = 0.318) was not independently and significantly related to the BI score at discharge.

**Conclusions:**

Increased intramuscular adipose tissue of the quadriceps at admission is more strongly and negatively related to ADL recovery at discharge than the loss of muscle mass among older patients with aspiration pneumonia. Interventions targeting the intramuscular adipose tissue of the quadriceps may improve ADL among these patients.

## Highlights

1. We determined the relationship between intramuscular adipose tissue at admission and ADL recovery at discharge in patients with aspiration pneumonia.

2. Quadriceps echo intensity at admission was negatively related to the Barthel Index score at discharge.

3. Quadriceps thickness at admission was not related to the Barthel Index score at discharge.

4. Increase in intramuscular adipose tissue of the quadriceps at admission is negatively related to the recovery of ADL at discharge in patients with aspiration pneumonia.

## Background

Aging causes loss of muscle mass, and the annualized rate of loss of leg muscle mass has been reported to be 1% in older people aged over 70 years [1]. In addition, sarcopenia is diagnosed by the presence of low muscle mass and muscle strength or physical performance [2] and is related to falls [3], low activities of daily living (ADL) [4], and mortality [5]. On the other hand, aging has also been reported to increase intramuscular adipose tissue [6]. An increase in the intramuscular adipose tissue is more strongly associated with decreased muscle strength [7, 8] and gait ability [9] and increased mortality [10, 11] than the loss of muscle mass. Additionally, an increase in the intramuscular adipose tissue of the quadriceps of older patients decreases ADL [12–14] and the swallowing ability [15] and is related to hospital-associated complications [16] more so than the loss of muscle mass. Therefore, an increase in the intramuscular adipose tissue of the quadriceps is considered a severe problem for older individuals.

Aspiration pneumonia occurs more frequently and is more severe among older individuals [17]. A previous study [18] reported that the decrease in ADL among patients with aspiration pneumonia is remarkable and that improving ADL among these patients is difficult. Because increased intramuscular adipose tissue of the quadriceps of older patients negatively affects ADL recovery more so than the loss of muscle mass [12–14], it is considered strongly related to ADL recovery among such patients. However, whether intramuscular adipose tissue of the quadriceps of older patients with aspiration pneumonia is related to ADL recovery after hospitalization remains unclear. Therefore, it is important to investigate this relationship to create an effective approach to improving ADL among older patients with aspiration pneumonia. This study aimed to determine the relationship between the intramuscular adipose tissue of the quadriceps at admission and ADL recovery at discharge among older patients with aspiration pneumonia.

## Methods

### Participants and study design

Older patients with aspiration pneumonia who were referred to the Department of Rehabilitation at Kasei Tamura Hospital in Japan participated in this prospective study. Aspiration pneumonia was diagnosed based on symptoms (fever, phlegm, and cough), inflammatory markers, chest imaging (including X-rays and computed tomography) results, and swallowing conditions. Patients younger than 65 years of age, insufficient data, and hospital admission because of other diseases were the exclusion criteria of this study. In addition, patients dying during the hospital stay due to aspiration pneumonia were also excluded. We initially enrolled a total of 455 patients; of these, 33 patients younger than 65 years of age, 18 patients with insufficient data, 363 patients admitted to the hospital because of the onset of other diseases, and two patients dying during the hospital stay due to aspiration pneumonia were excluded. Ultimately, this study included 39 patients diagnosed with aspiration pneumonia. The mean age of the participants was 83.5 years (± 7.6 years). Rehabilitation therapy, including physical therapy, occupational therapy, and speech and swallowing therapy, was performed for all participants during the hospital stay. In physical and occupational therapies, basic movement practices and joint range of motion exercises were performed. Resistance training was not sufficiently provided because this training is high intensity.

### Outcome measurements

The main outcome of this study was ADL at discharge. Characteristics of the participants, including age, sex, body weight, height, body mass index (BMI), intramuscular adipose tissue and muscle mass of the quadriceps, subcutaneous fat mass of the thigh, swallowing ability, nutritional status, inflammation, comorbidities, number of medications, number of rehabilitation therapy units (1 unit of rehabilitation therapy = 20 min), and ADL were determined within 72 h of admission. ADL were assessed at discharge as well as at admission. The length of the hospital stay (in days) and the number of days from the onset of the disease were determined at discharge. The hospitalization period at Kasei Tamura hospital was used to evaluate the length of the hospital stay. Eleven of the 39 patients were initially admitted to other hospitals; therefore, the lengths of both hospital stays were summed up and used as the number of days from the onset of disease. The participants in this study were a mix of patients immediately after the onset of aspiration pneumonia and those who had received acute care at other hospitals.

### Measurement of the recovery of activities of daily living

The Barthel Index (BI) [19] was used to assess ADL. ADL recovery during the hospital stay was evaluated using the BI score at discharge. The BI is an ordinal assessment with scores ranging from 0 to 100 points [19]. Lower BI scores indicate poor ADL. The BI consists of 10 items: feeding, moving from a wheelchair to a bed and from a bed to a wheelchair, grooming, toilet activity, bathing, walking on a level surface, going up and down stairs, dressing, bowel continence, and bladder continence [19].

### Measurements of the intramuscular adipose tissue and muscle mass in the quadriceps and subcutaneous fat mass in the thigh

A B-mode ultrasound system (NanoMaxx; SonoSite Japan, Tokyo, Japan) with a linear array probe (L25n/13–6 MHz; Nanomaxx; SonoSite Japan) was used to obtain transverse ultrasound images. We assessed the intramuscular adipose tissue and muscle mass of the rectus femoris and vastus intermedius based on the echo intensity and muscle thickness [6–9, 12–16, 20]. Previous studies using magnetic resonance imaging [21–23] reported the validity of intramuscular adipose tissue and muscle mass measurements using ultrasound. Ultrasound images of the rectus femoris and vastus intermedius were captured at 30% of the distance from the anterior superior iliac spine to the proximal end of the patella [6, 9, 12–15, 20]. Participants were in the supine position, with their lower limbs relaxed during imaging. We applied a water-soluble transmission gel to the skin surface of the thigh. To avoid muscle deformation, the probe was lightly pressed against the skin. The same investigator recorded all ultrasound images. Echo intensity was evaluated in the regions of interest selected to include as much muscle as possible while avoiding the bone and surrounding fascia [6, 9, 12–15, 20]. To standardize all echo intensity assessments, the gain was unified with the initial settings of the ultrasound system. Additionally, we used a uniform imaging depth of 60 mm for all echo intensity and muscle thickness measurements. The distance between the superficial adipose tissue–muscle interface and deep muscle–muscle interface was used to assess the thickness of the rectus femoris [6, 9, 12–15, 20]. The distance between the superficial muscle–muscle interface and bone–muscle interface was used to assess the thickness of the vastus intermedius [6, 9, 12–15, 20]. ImageJ 1.49 software (National Institutes of Health, Bethesda, MD, USA) was used to measure echo intensity and muscle thickness [6–9, 12–16, 20]. Echo intensity was assessed based on a computer-assisted 8-bit grayscale analysis and rated from 0 (black) to 255 (white) [6–9, 12–16, 20]. Greater intramuscular adipose tissue was indicated by higher echo intensity [24].

The mean echo intensity values of the rectus femoris and vastus intermedius were used as the quadriceps echo intensity during this study. We used the mean echo intensity values of the right and left quadriceps for the analysis. The sum of the thicknesses of the rectus femoris and vastus intermedius was treated as the quadriceps thickness. We used the mean thicknesses of the right and left quadriceps for the analysis. The measurement methods used to determine the echo intensity and muscle thickness of the rectus femoris and vastus intermedius have high reliability (intraclass correlation coefficient [1.1] = 0.857–0.959) [20]. Subcutaneous fat mass in the thigh was evaluated using the subcutaneous fat thickness. The distance between the dermis–adipose tissue interface and muscle–adipose tissue interface was used to determine the subcutaneous fat thickness of the thigh [6, 9, 12–15, 20]. We used the mean subcutaneous fat thicknesses of the right and left thighs for the analysis.

### Other characteristics measurements

The Food Intake Level Scale (FILS) [25], which is a 10-point observer-rated scale, was used to evaluate the swallowing ability. Better swallowing ability was indicated by higher FILS scores. We used the Geriatric Nutritional Risk Index (GNRI) score [26] to evaluate the nutritional status. The GNRI score was calculated using the following formula: GNRI score = (14.89 × serum albumin [g/dL]) + (41.7 × actual body weight [kg]/ideal body weight) [26]. The C-reactive protein level was used to assess the inflammatory status. Comorbidities were assessed based on the updated Charlson comorbidity index score [27].

### Statistical analysis

SPSS version 28 software (IBM SPSS Japan, Tokyo, Japan) was used for all statistical analyses. The normality of the variables was assessed using the Shapiro–Wilk test. Parametric and nonparametric data are presented as the mean ± standard deviation and median (interquartile range [IQR]), respectively. Nominal data are expressed as numbers and percentages.

The BI scores at admission and discharge were compared using the Wilcoxon signed-rank test. The Kendall rank correlation coefficient was used to assess the relationship between the quadriceps echo intensity and thickness and BI score at discharge. A partial correlation analysis that adjusted for the BI score at admission and subcutaneous fat thickness of the thigh was performed to determine the relationship between the quadriceps echo intensity and BI score at discharge. To assess the relationship between the quadriceps thickness and BI score at discharge during the partial correlation analysis, we used the BI score at admission as the control variable. A multiple linear regression analysis (forced entry method) was performed to confirm whether the quadriceps echo intensity was related to the BI score at discharge, even after adjusting for confounding factors. The age, sex, length of the hospital stay, quadriceps echo intensity and thickness, subcutaneous fat thickness of the thigh, FILS score, GNRI score, C-reactive protein level, updated Charlson comorbidity index, number of medications, number of rehabilitation therapy units, and BI score at admission were used as independent variables. During the multiple linear regression analysis, males and females were coded as 1 and 2, respectively. If the variance inflation factor was more than 10, then multicollinearity was considered. The subcutaneous fat thickness influences the echo intensity [28]. Based on this finding, we treated the subcutaneous fat thickness of the thigh as a confounding factor during the partial correlation and multiple regression analyses. *P* < 0.05 was considered statistically significant. Furthermore, the effect size (f^2^) of the multiple linear regression analysis was calculated using the following equation: R^2^/(1 − R^2^) [29]. The statistical power of the multiple linear regression analysis was calculated based on f^2^, an alpha error of 0.05, the total sample size, and the number of predictor variables. G^*^ Power version 3.1.9.2 (Heinrich-Heine-Universität Düsseldorf, Düsseldorf, Germany) was used to calculate the statistical power.

### Sample size calculation

According to a recent prospective study [13], the effect size (f^2^) of the multiple linear regression analysis of the BI score at discharge was 2.049 (during this multiple regression analysis, the quadriceps echo intensity and six additional variables were independently and significantly associated with the BI score at discharge of older patients). During this study, a similar effect size in the multiple linear regression analysis of the BI score at discharge was expected. The a priori sample size calculation based on the effect size (f^2^) of 2.049, power of 0.95, alpha error of 0.05, and 13 to 15 predictors indicated that a sample size of at least 27 to 30 participants was required. We used G*Power version 3.1.9.2 to calculate the sample size.

## Results

The median BI scores at admission and discharge were 15.0 (IQR, 0.0–35.0) and 20.0 (IQR, 5.0–55.0), respectively. The BI score at discharge was significantly higher than that at admission (*p* = 0.002). The median number of days from the onset of disease and the median length of the hospital stay were 77.0 (IQR, 49.0–93.0) days and 70.0 (IQR, 37.0–92.0) days, respectively. Characteristics of the participants at admission are presented in Table 1. Table 2 shows the results of the correlation and partial correlation analyses. The results of the correlation analysis indicated that the quadriceps echo intensity was significantly and negatively related to the BI score at discharge and that the quadriceps thickness was significantly and positively related to the BI score at discharge. The results of the partial correlation analysis indicated that although the quadriceps echo intensity was significantly and negatively related to the BI score at discharge, the quadriceps thickness was not significantly related to the BI score at discharge.

**Table 1:** Characteristics of participants at admission

Characteristics	
Age, years	83.5 ± 7.6
Sex, male/female	26 (66.7)/13 (33.3)
Height, cm	158.0 (149.0–164.0)
Body weight, kg	43.6 (35.2–47.5)
Body mass index, kg/m^2^	17.8 ± 3.2
Quadriceps echo intensity (grayscale range, 0–255), arbitrary units	92.7 ± 19.7
Quadriceps thickness, cm	1.0 ± 0.4
Subcutaneous fat thickness of the thigh, cm	0.3 ± 0.1
Food Intake Level Scale score	7.0 (6.0–7.0)
Serum albumin, g/dL	3.0 ± 0.4
Geriatric Nutritional Risk Index score	77.4 ± 9.0
C-reactive protein, mg/dL	1.2 (0.4–6.8)
Updated Charlson comorbidity index score	2.0 (2.0–3.0)
Number of medications	5.0 (3.0–8.0)
Number of rehabilitation therapy units, units/day	3.0 (2.0–4.0)
Barthel Index score	15.0 (0.0–35.0)

Data are presented as the mean ± standard deviation, n (%), or median (interquartile range)

**Table 2:** Relationships between echo intensity and muscle thickness of the quadriceps at admission and Barthel Index score at discharge

Variables	Barthel Index score at discharge	
Quadriceps echo intensity at admission	− 0.342^a^ (*p* = 0.003)	− 0.334^b^ (*p* = 0.043)
Quadriceps thickness at admission	0.274^a^ (*p* = 0.021)	0.066^c^ (*p* = 0.693)

^a^Kendall’s τ rank correlation coefficient. ^b^Partial correlation coefficient adjusted for the Barthel Index score at admission and subcutaneous fat thickness of the thigh. ^c^Partial correlation coefficient adjusted for the Barthel Index score at admission

Table 3 shows the result of the multiple linear regression analysis of BI scores at discharge. No multicollinearity was observed between the independent variables. The quadriceps echo intensity (β =  − 0.374; *p* = 0.036) and BI score at admission (β = 0.601; *p* < 0.001) were independently and significantly related to the BI score at discharge (R^2^ = 0.718; f^2^ = 2.546; statistical power = 1.000). The quadriceps thickness (β =  − 0.216; *p* = 0.318) was not independently and significantly related to the BI score at discharge.

**Table 3:** Multiple regression analysis of the Barthel Index score at discharge

	B	SE	95% Confidence interval of B	β	VIF	*p*-value
Age	0.302	0.463	− 0.651, 1.256	0.081	1.353	0.520
Sex	2.216	8.064	− 14.393, 18.825	0.037	1.637	0.786
Length of the hospital stay	0.085	0.103	− 0.127, 0.297	0.113	1.642	0.415
Quadriceps thickness at admission	− 15.238	14.962	− 46.052, 15.575	− 0.216	3.997	0.318
Quadriceps echo intensity at admission	− 0.538	0.243	− 1.038, − 0.038	− 0.374	2.527	0.036
Subcutaneous fat thickness of the thigh at admission	19.429	39.764	− 62.468, 101.325	0.081	2.430	0.629
Food Intake Level Scale score	3.454	2.167	− 1.010, 7.917	0.245	2.105	0.124
Geriatric Nutritional Risk Index score	0.436	0.530	− 0.656, 1.528	0.138	2.511	0.419
C-reactive protein	− 0.178	0.645	− 1.506, 1.150	− 0.033	1.235	0.785
Updated Charlson comorbidity index score	1.342	2.086	− 2.955, 5.639	0.076	1.232	0.526
Number of medications	− 1.077	0.899	− 2.928, 0.775	− 0.150	1.398	0.242
Number of rehabilitation therapy units	− 3.106	2.775	− 8.822, 2.610	− 0.152	1.634	0.274
Barthel Index score at admission	0.784	0.177	0.419, 1.149	0.601	1.638	< 0.001

B, partial regression coefficient, *SE* Standard error, *β* Standardized partial regression coefficient, *VIF* Variance inflation factor

## Discussion

This study examined the relationship between the intramuscular adipose tissue of the quadriceps at admission and ADL recovery at discharge among older patients with aspiration pneumonia. The results of our study indicate that an increase in intramuscular adipose tissue of the quadriceps is more strongly and negatively related to ADL recovery than the loss of muscle mass among older patients with aspiration pneumonia.

Recently, it was reported that an increase in the intramuscular adipose tissue of the quadriceps in older patients negatively affects ADL recovery more so than the loss of muscle mass [13]. Our results support the results of that study [13]. Furthermore, recent studies reported that the intramuscular adipose tissue of the quadriceps in older patients is more strongly related to swallowing ability [15] and hospital-associated complications [16] than muscle mass. Additionally, in some studies, the muscle mass values included intramuscular adipose tissue as well as actual muscle mass, which overestimates muscle mass [10, 30].

A BI score < 60 indicates a severely dependent status [31]. In the present study, the median BI score of the participants at admission was 15.0 (IQR, 0.0–35.0), which is very low. Another study reported that BI scores of older patients with aspiration pneumonia were also very low (the mean BI scores of the early rehabilitation and no rehabilitation groups were 14.6 and 18.9, respectively) [18]. Although the median BI score at discharge (20.0 [IQR, 5.0–55.0]) was statistically and significantly higher than that at admission (15.0 [IQR, 0.0–35.0]) in our study, a major improvement was not observed. Similarly, it has been reported that among older patients with aspiration pneumonia, changes in the BI score during the hospital stay were less than 0 for most of them (71.1%), and their ADL were less likely to improve [18]. The mean age of the participants in that study was 83.9 years (± 8.3 years) [18], and that of our participants was 83.5 years (± 7.6 years). Additionally, some studies [18, 32] reported that many patients with aspiration pneumonia have low BMI. The Global Leadership Initiative on Malnutrition criteria indicated that low BMI is < 20 kg/m^2^ in Asian populations older than 70 years [33]. Based on this criterion, the BMI of most patients (76.9%) in our study was also low. Therefore, it was considered that the participants in the present study properly represented older patients with aspiration pneumonia.

Early rehabilitation improves ADL among older patients with aspiration pneumonia more effectively than no rehabilitation [18]. Therefore, rehabilitation is important for improving ADL among such patients. Based on our results, it may be important to perform interventions targeting the intramuscular adipose tissue of the quadriceps to improve ADL among older patients with aspiration pneumonia. Resistance training can effectively improve intramuscular adipose tissue of the quadriceps of older adults [34, 35]. However, the mean GNRI score of the participants in the present study was 77.4 (± 9.0). A GNRI score < 82 and GNRI scores ranging from 82 to 92 indicate major and moderate malnutrition risks [26], respectively; therefore, the nutritional status of almost all participants in the present study was poor. Resistance training is considered difficult for older patients with aspiration pneumonia, a low nutrition status, and poor ADL, such as those in the present study. However, physical activity and nutritional interventions can effectively improve the intramuscular adipose tissue of the thigh in older adults with mobility limitations [36]. Therefore, interventions comprising physical activity other than resistance training are considered realistic for older patients with aspiration pneumonia who want to improve their ADL.

Influences of aging and disuse on the loss of muscle mass in the quadriceps are remarkable among the upper and lower extremities muscles [37, 38]. It has been reported that muscle mass of the quadriceps in critically ill patients is more strongly predicted for mortality than the appendicular skeletal muscle mass measured by bioelectric impedance [39], and the decrease of intramuscular adipose tissue of the quadriceps of older inpatients who were not able to walk independently is more strongly related to the improvement of gait independence than the loss of muscle mass [40]. In addition, the decrease of intramuscular adipose tissue of the quadriceps is closely related to the recovery of swallowing ability of older inpatients who had severely dependent statuses in ADL [15]. Furthermore, using the quadriceps thickness to assess muscle mass has been recommended by the International Society of Physical and Rehabilitation Medicine to diagnose sarcopenia [41]. Based on these findings, although the participants of the present study were almost all in severely dependent conditions in ADL, targeting the quadriceps for determining the relationships between intramuscular adipose tissue and muscle mass and ADL recovery was considered valid.

Fukumoto et al. [42] reported that cut-off values for quadriceps thickness to detect low skeletal muscle mass index of older males and females were 2.88 cm and 2.34 cm, respectively. The mean quadriceps thickness of our study was 1.0 cm (± 0.4 cm), and it was smaller than the cut-off values of the study by Fukumoto et al. [42]. However, Fukumoto et al. [42] determined the quadriceps thickness at the mid-point of the distance from the greater trochanter to the lateral condyle of the femur. On the other hand, our study captured the quadriceps thickness at 30% of the distance from the anterior superior iliac spine to the proximal end of the patella. Although the measurement point of quadriceps thickness was different between our study and that of Fukumoto et al. [42], quadriceps thickness in our study was considered to be thinner than the cut-off values of the study by Fukumoto et al. The cut-off values of quadriceps echo intensity to detect low muscle function as assessed by handgrip strength and gait speed in older males and females have been reported to be 41.7 and 44.8, respectively [43]. Although comparing the echo intensity between ultrasound devices is difficult because it fluctuates by the system of the ultrasound and the angle of the probe [44], the mean quadriceps echo intensity in our study was 92.7 (± 19.7), and it was higher than the abovementioned cut-off values. Taken together, the muscle conditions of the participants in our study were considered to be poor.

This study had some limitations. First, we did not measure quadriceps echo intensity and thickness at discharge. Therefore, whether changes in quadriceps echo intensity and thickness in older patients with aspiration pneumonia are related to recovery of ADL remains unclear. In the future, a longitudinal study will be required. Second, the mean BMI of the participants in the present study was very low (17.8 ± 3.2 kg/m^2^). Therefore, many participants were considered to be sarcopenic or cachectic. However, we were unable to present a prevalence rate of sarcopenia or cachexia because we did not measure muscle function. Finally, the sample size of this study was small. However, the statistical power of the multiple regression analysis of the BI score at discharge was 100%. Therefore, there were no type 2 errors in the multiple linear regression analysis during this study.

## Conclusions

Increased intramuscular adipose tissue of the quadriceps at admission is more strongly and negatively related to ADL recovery at discharge than the loss of muscle mass among older patients with aspiration pneumonia. Interventions targeting the intramuscular adipose tissue of the quadriceps may improve ADL among these patients.

## Data Availability

All data generated or analyzed during this study are included in this published article.

## Abbreviations

ADL: Activities of daily living
BMI: Body mass index
BI: Barthel index
FILS: Food intake level scale
GNRI: Geriatric nutritional risk index
